# A blockchain-based lightweight identity authentication scheme for the IEDs of security and stability control system

**DOI:** 10.1371/journal.pone.0265937

**Published:** 2022-03-24

**Authors:** Zhaohan Li, June Li, Siyu Zhao, Xiong Chen, Ke Feng, Wang Wang

**Affiliations:** 1 Key Laboratory of Aerospace Information Security and Trusted Computing, Ministry of Education, School of Cyber Science and Engineering, Wuhan University, Wuhan, China; 2 State Grid Electric Power Research Institute, Nanjing, China; University College of Engineering Tindivanam, INDIA

## Abstract

As an important part of the second defense line of the power system, the Security and Stability Control System (SSCS) is of great significance to ensure the reliable operation of the power system. However, SSCS still lacks an effective security mechanism and is easily accessed by attackers, thus posing a threat to the stable and reliable operation of the power system. To tackle this issue, we propose a blockchain-based identity authentication scheme for Intelligent Electronic Devices (IEDs) of SSCS. We first propose an identity authentication system model for IEDs and design the deployment of consortium chain nodes on IEDs, with architectural characteristics of SSCS and the working scenario of IEDs taken into consideration. The consortium chain is used to store credentials required for authentication, ensuring that they are tamper-proof. We combine IP address, port number and physical ID, and propose the unique identification of IEDs, with a data structure designed for the identification. We also propose a lightweight identity authentication method based on renewable hash chains, with hash chains used as one-time authentication passwords, and introduce a renewal mechanism of hash chains. Further, the detailed processes of registration and authentication phase are designed. Finally, the security analysis shows that our identity authentication scheme can resist various attacks, and the feasibility of our scheme is verified by experiments.

## 1. Introduction

**Fig 1:**
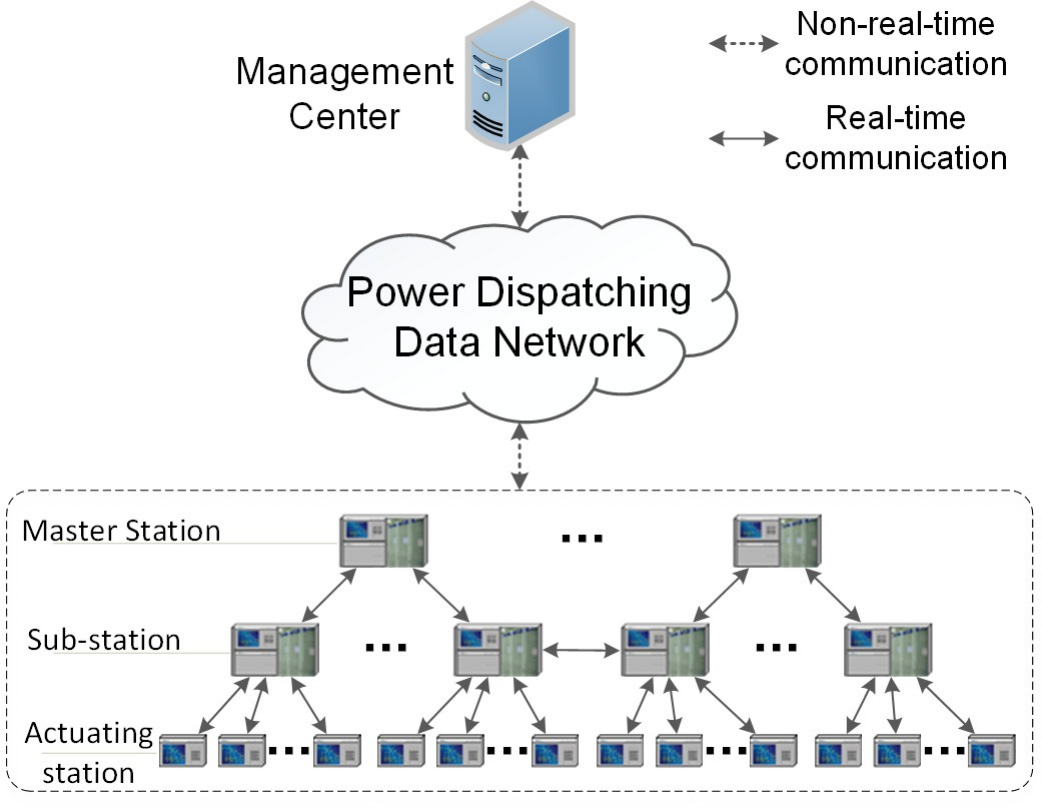
Layered architecture of SSCS.

To reduce losses when encountering accidents and ensure the reliable and stable operation of the power system, the concept of three-defense lines has been proposed in China [1]. As an important part of the second defense line, the Security and Stability Control System (SSCS) [1,2], also known as System Protection Scheme (SPS) [3], remedial action scheme (RAS) [4] and Wide Area Protection System (WAPS) [5], is used to ensure the reliable and stable operation of the power system in the event of a small probability of a serious failure. As shown in Fig 1, SSCS is generally deployed vertically in multiple layers, with different functions in each layer. Master stations on the top layer are responsible for decision making and command issuance of SSCS, while collecting the status information of the whole network through sub-stations at the same time. Sub-stations are responsible for sending local load information, unit information, operation and shutdown status of contact elements, and all kinds of failure information to master stations, and receiving commands from master stations. On the lowest layer of SSCS, actuating stations are responsible for monitoring and sending local load and power generation information to sub-stations, as well as receiving commands of generator tripping, load shedding and power generating from sub-stations [6]. Undertaking more complex services, IEDs of master stations tend to have more computing and storage resources than those of sub-stations and actuating stations.

To realize centralized monitoring and management of IEDs in SSCS, the Management Center (MC) is deployed in the power dispatching central station and is responsible for data collection and real-time monitoring of the IEDs, real-time alarm, historical data storage management and so on. The IEDs communicate with each other in real-time through dedicated optic fiber channels, and at the same time carry out non-real-time communication with MC via the power dispatching data network. Since the dedicated communication channels between IEDs have limited bandwidth and the connections are point-to-point, non-control services communication between IEDs, such as sending or receiving authentication requests, can be carried out via the power dispatching data network, with MC as a relay.

In recent years, to realize the efficient utilization of energy and meet various needs of electrical energy consumers, a growing number of intelligent terminals have been connected and interconnected in the power system. The increasing operational complexity and expanding new functions of the power system put forward higher requirements for the reliability and flexibility of SSCS, and also make it easy for adversaries to find more ways to carry out cyber attacks. However, SSCS is still lacking in effective security mechanisms currently and would be easily accessed by adversaries illegally, thus posing a threat to the safe and stable operation of the power system.

As the basis of other security mechanisms, identity authentication mechanism can be the key to protecting SSCS from the threat of illegal access. Only when effective identity authentication is realized can other security mechanisms, such as access control, security audit and intrusion detection, be better implemented. A certificate management system based on Public Key Infrastructure (PKI) has been deployed in the existing power system and is responsible for the communication security of the power dispatching data network [7,8]. However, it is not appropriate to use certificates for the identity authentication of Intelligent Electronic Devices (IEDs) as well, which are the main components of SSCS. On the one hand, certificate authentication will bring a computational burden to IEDs with limited computing and storage resources. On the other hand, authentication based on certificates relies a lot on Certificate Authorities (CAs), which are the core components of PKI and are vulnerable to attacks leading to a single point of failure. To tackle this issue, we consider that a lightweight and distributed identity authentication scheme should be designed for IEDs of SSCS.

As a decentralized ledger with inherently distributed characteristics, blockchain seems to provide a new solution for the identity authentication of IEDs that meets our expectations. Blockchain arranges blocks in chronological order and ensures that data stored in the blocks are tamper-proof and unforgeable through underlying cryptographic algorithms [9]. Furthermore, the consensus mechanism of blockchain enables multiple nodes to achieve mutual trust without deploying trusted third-party institutions or trusted centers. Applying blockchain to the identity authentication of IEDs can avoid additional system deployment overhead that PKI-based authentication schemes may cause and meet the security requirements of SSCS.

In this paper, taking the characteristics of SSCS into consideration, we propose a blockchain-based lightweight distributed authentication scheme for IEDs, which achieves mutual trust among IEDs and improves the ability of SSCS to cope with cyber attacks.

The major contributions of this paper are as follows:

1. Considering architectural the characteristics of SSCS and the working scenario of IEDs, we propose an identity authentication system model for the IEDs and design the deployment of consortium chain nodes on IEDs, thus realizing the decentralized identity authentication of IEDs.
2. We combine IP address, port number and physical ID (pID) as the unique identification of IEDs, with a data structure designed for the identification. The unique identification represents both the cyber identity and the life-cycle traceable physical identity of IEDs. Therefore, it can be used as a communication address as well as a credential for achieving the traceability of IEDs.
3. We propose an efficient and lightweight identity authentication method. The proposed method uses hash chains as one-time authentication passwords and requires only one time hash calculation in the authentication process without the need to deploy any third party for key management. We also design an automatic regeneration method of the hash chain, which realizes the continuity of the identity authentication.
4. We design the processes of registration and authentication of IEDs, which makes the scheme more suitable for SSCS and makes it simple for other researchers to understand.
5. We propose a timeout mechanism to increase the security of our scheme. This mechanism together with the one-way nature of the hash function make our authentication scheme achieve unforgeability.

The rest of this paper is organized as follows. In Section 2, we review related work of authentication schemes for resource-strained systems and devices. The introductions of physical ID and blockchain as well as the system model and the threat model of the proposed scheme are given in Section 3. Then, in Section 4, we separately illustrate the renewable identity hash chain, which is the main mechanism of our authentication method. In Section 5, lightweight identity authentication scheme for IEDs of SSCS based on blockchain is presented in detail, while the processes of registration and authentication are designed. In Section 6, security theorems of the proposed scheme are given and proved. Section 7 shows the experimental results and analysis. Finally, we conclude our work and propose the future plan in Section 8.

## 2. Related work

For resource-constrained systems like SSCS and devices like IEDs, many authentication schemes have been proposed.Some researchers applied PKI-based certificate authentication mechanisms to the power system [10–12]. Liu et al. [10] introduced a distributed certificate authentication system developed for the power dispatching system, which simplified the deployment of traditional PKI-based authentication mechanism. However, functions that a CA should have were still centralized on a trusted device, which could be attacked and cause a single point of failure. Luo et al. [11] improved the traditional PKI-based authentication mechanism and designed a security support platform for the power secondary system. The improved PKI system issued certificates supporting the SM2 algorithm instead of the most used RSA algorithm, thus improving the efficiency and security of authentication. Chen et al. [12] proposed CertChain, a blockchain-based public and efficient certificate audit scheme, which avoided centralization in practice and achieved CA-based trust disperse through designing a distributed dependability-rank-based consensus protocol. Although the schemes proposed in [11,12] have made improvements to traditional PKI-based authentication mechanisms, they still need to consider about certificate management, which can result in a consumption of system resources. Therefore, Saxena et al. [13] proposed an integrated protocol providing mutual authentication for multiple communication entities in a smart grid. The scheme used distributed cloud servers as trusted third-party authorities, replaced the traditional centralized PKI with a distributed key generation center, and implemented a certificate-free message authentication code authentication method. However, the large number of distributed cloud servers increases deployment costs, thus making it difficult to be applied to resource-constrained systems.

Some researchers have also proposed authentication and key agreement protocols based on the Elliptic Curves Cryptography (ECC) algorithm for resource-constrained systems [14–18]. Mahmood et al. [14] proposed a mutual authentication scheme between smart appliances and substations. The use of ECC algorithm with short keys and high security made the proposed scheme resistant to all known security attacks while having low computational and communication costs. Compared with the scheme in [14], the authentication protocol Wang et al. [15] proposed for edge computing-based smart grid system used public key instead of users’ real identity ID for authentication to ensure anonymity, while preventing user behavior from being traced by blinding the public key to provide unlinkability. It also introduced blockchain to handle key issuing, updating, and revocation with higher security. Whereas, schemes in [14,15,18] all require a trusted third party for key generation and management, which complicates the system deployment. Moreover, authentication protocols presented in [14–18] need to complete the authentication and key agreement process before entities start to communicate, which is not compatible with the working scenario of continuous communication of IEDs in SSCS.

Considering protecting the physical security of resource-constrained devices, anonymous authentication schemes based on Physical Unclonable Functions (PUFs) were proposed [19,20]. These schemes use lightweight cryptographic primitives such as PUFs and one-way hash functions that do not require any sensitive information (e.g., keys) to be stored on resource-constrained devices, reducing overhead while improving security.

There are also researchers presented lightweight authentication protocols using lossless compression algorithms and Merkle trees to compress data, which reduced the computational, communication, and storage costs while improving the confidentiality of data [21]. However, this scheme can only efficiently reduce various costs when it is applied to scenarios where a large amount of data are transmitted during communication, and the amount of data transmitted by IEDs during communication is inherently small, which can not well reflect the lightweight characteristics of this scheme.

As trustworthiness evaluation of devices is an important constituent of data source authentication, Xia et al. [22] designed a cloud-aided trustworthiness evaluation mechanism and proposed an anonymous authentication and key aggreement scheme based on non-interactive zero knowledge argument for the problem of easy leakage of user privacy in the authentication process. Although the scheme has better performance compared with other similar schemes while ensuring the privacy and data security of IoT devices, the deployment of cloud servers can bring additional overhead to some resource-constrained systems.

In addition, with increasing attention fascinated to the security of Vehicular Ad-hoc Networks (VANETs), some researchers have proposed lightweight authentication protocols and handover authentication methods for resource-constrained vehicles and Roadside Units (RSUs) [23–26]. Wang et al. [23] proposed a lightweight authentication protocol for timely avoidance of Emergency Vehicles (EVs). After completing the first authentication with the nearest RSU, an EV can complete the mutual authentication with the subsequent RSUs by providing only some simple parameters, thus reducing the computational overhead and improving the authentication efficiency. Azees et al. [24] combined the distributed nature of VANETs and blockchain to present an anonymous authentication scheme to validate the legitimacy of vehicle users, and also proposed an anonymous handover authentication method, which enabled the authentication information of a vehicle to be transferred between different RSUs and reduced the overhead caused by repeated authentication. Blockchain is used to ensure the security of the authentication codes of vehicle users and realize the traceability of disobedient vehicles. As authentication with unlinkability is one of the critical requirements for the security of VANETs, Liu et al. [25] proposed a blockchain-based unlinkable authentication protocol, making dispersed service managers constitute a distributed data sharing database. Vehicles are able to use self-generated multiple pseudonyms associated with their real identities to prove the legitimacy of their identities to the service managers, which realizes the traceability of the vehicle identity while ensuring the unlinkability. Vijayakumar et al. [26] proposed a dual authentication scheme, which used two factors, vehicle secret key and user fingerprint, to authenticate the vehicles and effectively resist replay attack and masquerade attack, and also proposed a corresponding efficient group key management mechanism, whose computational complexity and communication complexity both achieved *O*(1). However, these authentication schemes require trusted third-party authorities to verify the identities of vehicles or RSUs or to maintain the blockchains, which does not fit well with the peer-to-peer authentication scenario of IEDs in SSCS.

To sum up, there are various authentication schemes proposed for resource-constrained systems and devices, though, none of them are designed considering the working scenario of IEDs of SSCS. That is to say, our work of designing a lightweight and distributed identity authentication for IEDs of SSCS is of great significance.

## 3. Preliminaries

### 3.1 Identity code for power grid assets

**Fig 2:**
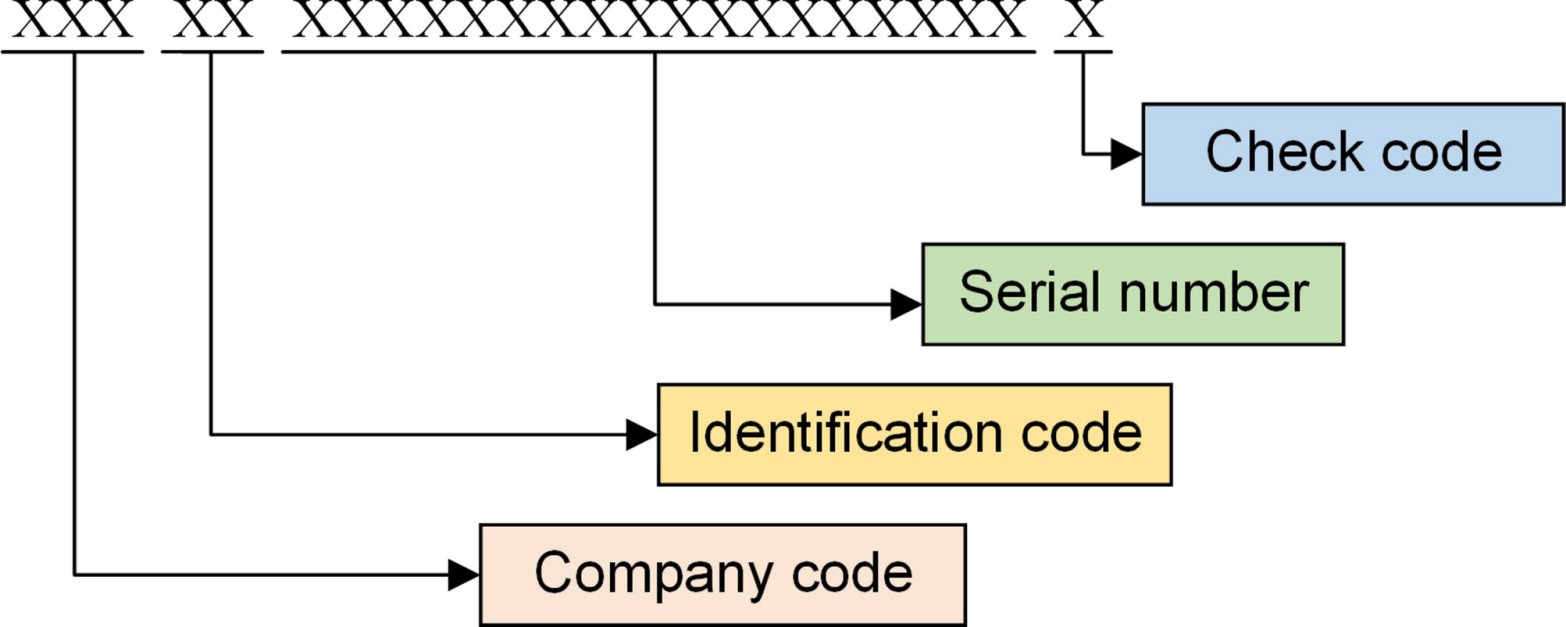
Composition of pID.

All physical assets under the jurisdiction of power grid enterprises in China, including power primary equipment, power secondary equipment, power marketing equipment, etc., are collectively referred to as power grid assets. Electronic Product Code (EPC) is widely used in Europe and the United States for unified coding of information and communication assets at present [27]. Similarly, to realize the multi-code linkage as well as information integration of various professional codes such as project code, work breakdown structure code, material code, equipment code, and asset code in the process of power grid asset management, power grid enterprises in China proposed and introduced a unified identity code for power grid assets (also called physical ID, i.e. pID) [28]. The pID is generated in stages of procurement or operation monitoring of equipment, with a two-dimensional code or Radio Frequency Identification (RFID) electronic tag as the carrier, and is the only lifetime identity code of a power grid asset. A pID is generally in form of a 24-digit decimal number containing company code, identification code, serial number and check code, and the composition of pID is shown in Fig 2.

### 3.2 Blockchain and consensus mechanism

**Fig 3:**
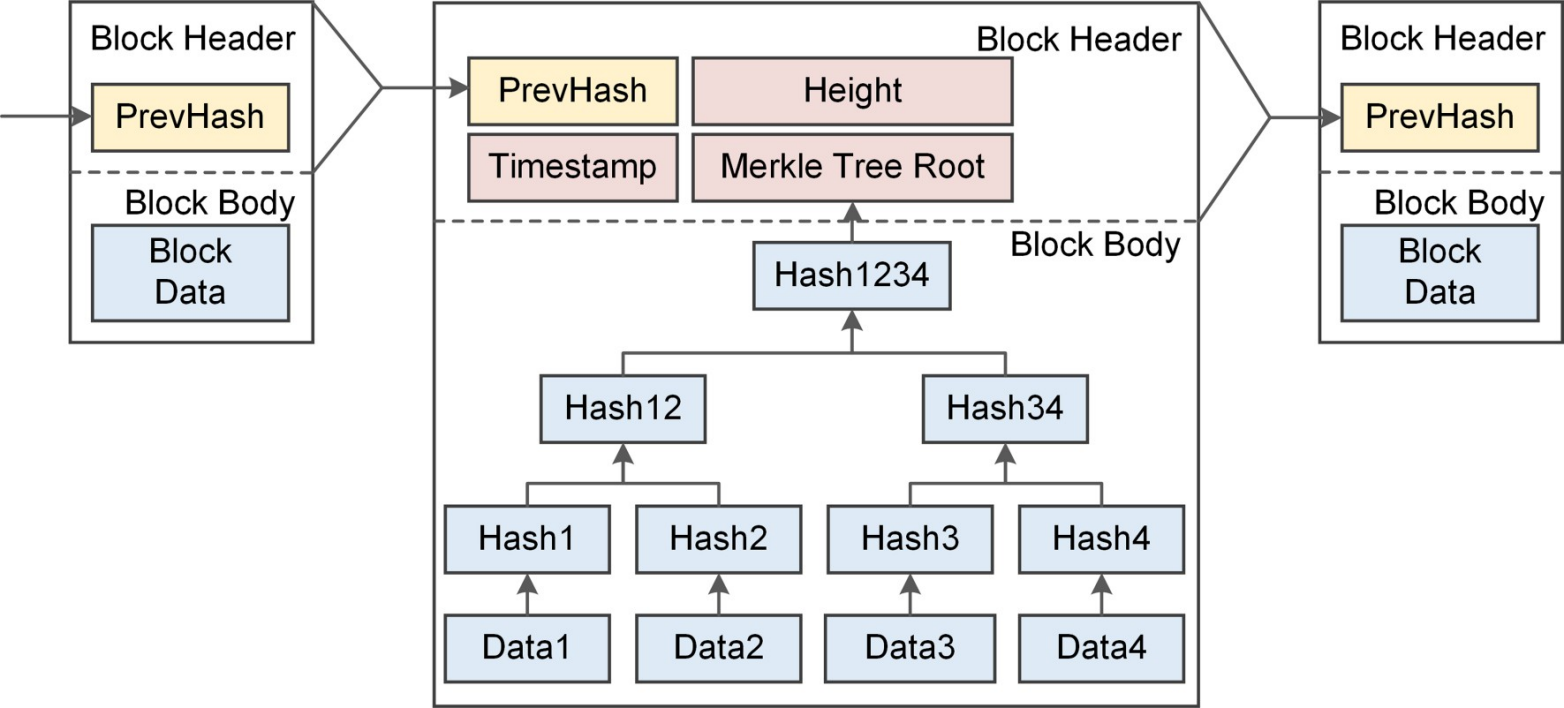
Block structure of the blockchain.

Blockchain is a chained data structure that connects blocks in chronological order and the structure of a block used in our scheme is depicted in Fig 3. Each block consists of a block header and a block body. Data in the block body is stored in the form of Merkle Tree to facilitate integrity checking. Blockchain constructs a self-organizing network in a peer-to-peer scenario and relies on underlying cryptography technologies to ensure that data on-chain is tamper-proof and unforgeable. The structure of the block header used in our scheme is shown in Table 1, which consists of Timestamp, Height, PrevHash and MerkleTreeRoot. The data stored in the block body are authentication credentials published by AEs, which consists of unique identification of IEDs, hash chain indexes and the hash value corresponding to the indexes.

**Table 1:** Fields of the block header.

Field	Description	Size
Timestamp	Timestamp of the block	8 bytes
Height	Number of blocks between the block and the genesis block	8 bytes
PrevHash	Hash of the previous block	32 bytes
MerkleTreeRoot	Root of the Merkle tree of authentication information	32 bytes

Blockchain can be divided into three catergories according to the degree of decentralization: public chain, consortium chain and private chain [29]. The public chain has no user authorization mechanism and is completely decentralized, thus any person or organization can freely access the network and participate in bookkeeping anonymously. The consortium chain is jointly managed and maintained by several organizations, which means it is paritially decentralized. Only the designated nodes authorized by the organizations can be the bookkeeper of the consortium chain. Similar to the consortium chain, the private chain is also a kind of permissioned chain, but a private chain is managed by only a single organization and is significantly less decentralized. Certain access thresholds and interest constraints among all the participants can ensure the high credibility of the permissioned chain without the need for a large node scale. Moreover, the consortium chain has better performance than the public chain and has a higher decentralization degree than the private chain, with high transaction speed, which make it more suitable for resource-contrained SSCS. Therefore, our identity authentication scheme is designed based on the consortium chain.

Blockchain relies on consensus algorithms to achieve consistency and correctness of ledger data among different nodes. The most commonly used consensus algorithms are Proof of Work (PoW), Proof of Stake (PoS), Delegated Proof of Stake (DPoS), Practice Byzantine Fault Tolerance (PBFT), etc. PoW, PoS and DPoS all rely on virtual currencies and certain incentive mechanisms to reach consensus, which can be resource-intensive when used in blockchain deployed for SSCS. Improved based on the Paxos algorithm, PBFT can handle Byzantine errors and can provide (n-1)/3 fault tolerance in an n-node system while guaranteeing both liveness and security [30,31]. Unlike PoW and other algorithms, PBFT reaches consensus through a voting mechanism, which can solve the forking problem and can improve efficiency at the same time. However, the voting mechanism needs to be interactively carried out by nodes in a closed cluster, which increases the communication complexity of nodes. Therefore, PBFT is mostly suitable for permissioned chains with small node scales. SSCS has a small number of master stations, which means the number of AuNs on the consortium chain is quite limited. Therefore, according to analysis in Section 3.3, we choose PBFT as the consensus algorithm of the consortium chain used in the proposed scheme, which has high operational speed on small-scale systems [32].

### 3.3 System model

**Fig 4:**
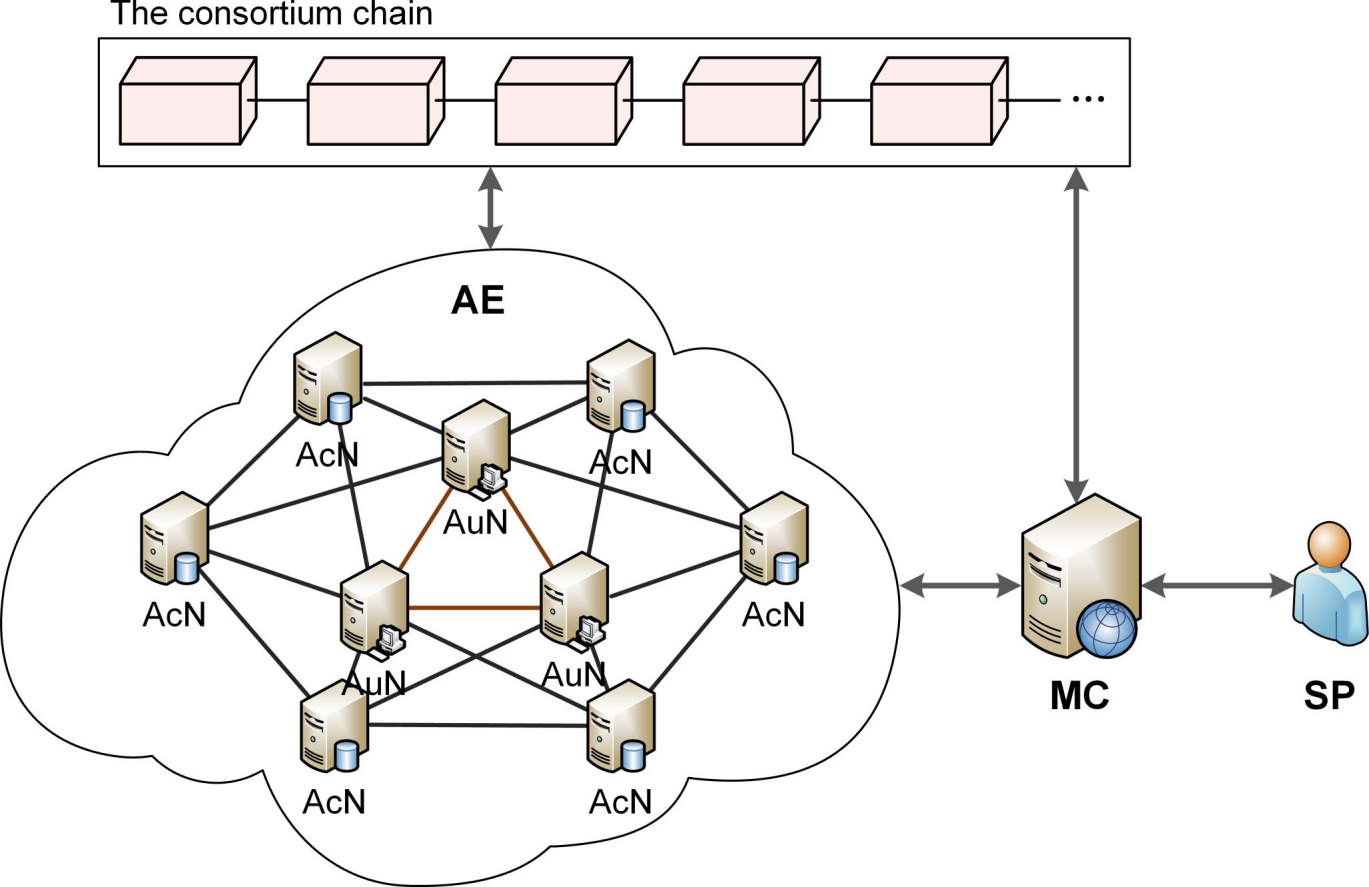
System model of identity authentication for IEDs of SSCS.

As shown in Fig 4, the system model for the proposed scheme consists of three major entities: Authentication Entity (AE), MC and Security Personnel (SP).

AEs correspond to IEDs of master stations, sub-stations and actuating stations in SSCS that has completed registration phase. All of the AEs are connected to the consortium chain network. Since according to Section 1, IEDs of master stations have more computing and storage resources than those of other stations, they are deployed as Authorized Nodes (AuNs) after join in the network, which participate in the consensus process and are responsible for maintaining the consortium chain. IEDs of sub-stations and actuating stations are deployed as Access Nodes (AcNs), which only have the authority for querying data on-chain. Point-to-point communication for authentication is conducted between any two AEs at certain intervals via the power dispatching data network. If an abnormal authentication result is received, the AE that requests for authentication will report an alert to MC.

MC is the administrator of SSCS, and is responsible for updating and verifying the communication relationship list of each AE, which records the information of AEs directly connected with this AE through dedicated communication channels. MC also joins the consortium chain network and participates in consensus process and chain maintenance.

SP are responsible for ensuring the credibility of MC, maintaining lists of communication relations of AEs, and timely handling the abnormal authentication results received by MC. At the same time, SP are in charge of managing the members of the consortium chain, and all AEs must get permission from SP before they join the consortium chain network. Moreover, SP are assumed to be fully trusted.

Our proposed scheme consist of two phases: registration phase and authentication phase. An IED needs to register before it joins the authetication system and becomes an AE. To ensure the initial security of an IED, the registration phase is mainly led by trusted SP and MC. In the registration phase, the IED needs to generate a unique identification, get its communication list, join the consortium chain network, generate its identity hash chains, and publish the initial authentication credential on the consortium chain. The consortium chain underlying the identity authentication system is applied to replace the traditional database to ensure the reliability and integrity of authentication credentials. Then, in the authentication phase, an AE send request to all other AEs that have conmmunication relationships with it in order to validate their identity successively. Once an authentication process is not passed, the identification of the relevate AE will be reported to MC as an alert and the security of the AE will be thoroughly checked.

### 3.4 Threat model

Since AEs communicate with each other over insecure channels in the proposed scheme when authenticating, we assume that an attacker (e.g., Dolce-Yao threat model [33]) can eavesdrop and intercept the exchanged messages during the two-way communication between AEs. An attacker can attempt to replay the messages he/she obtained to the communicating party that should have received them. He/She can also forges indentification and messages for own purpose. However, the attacker cannot easily obtain information stored in the local cryptographic chip on the AE. Under these premises, several possible attacks on SSCS are listed as follows:

#### Replay attack

The attacker may obtain the identification of a legitimate AE and the authentication message the AE currently send to other AE through eavesdropping and intercepting. Then he/she can modify and replay the identification and authentication password extracted from the message to another AE, thus disturbing the authentication process to make the eavedropped AE under suspicion, or directly replay them to pass the authentication, thus further carrying out masquerade attack.

#### Masquerade attack

The attacker can implement this attack after carrying out the replay attack. After replaying the identitfication and authentication password, and passing the authentication, he/she can masquerade as the eavesdropped legitimate AE. Or the attacker may also forge a legitimate identity by generating the identification based on the established rules. Anyway, the attacker can obtain a legitimate identity, and further realize the forged measurement information to be sent up or malicious control cmmands to be issued, causing miss-operation of the circuit and disturbing the stable operation of the system.

#### Denial of Services (DoS) attack

In this attack, the attacker can take some means to make entities in the system unable to provide services, such as sending a large number of authentication requests to a single AE and disabling it.

## 4 Renewable identity hash chain

### 4.1 Hash chain authentication principle

The concept of hash chains was first proposed by Lamport [34,35] and was designed to take advantage of the public key-like nature and high computational efficiency of hash chains to be used as One-Time Passwords (OTP) for identity authentication.

The identity hash chain of an AE is generated for the first time in the registration phase. When an authentication entity *A* needs to generate a hash chain of length *n*, *A* first selects a random number *S*_*A*_ as the seed, and then uses the hash function *h* selected by the scheme to compute *S*_*A*_ for *n* times recursively:

$$\left.\begin{array}{c} h^{1}\left(S_{A}\right)=h\left(S_{A}\right)\\ h^{2}\left(S_{A}\right)=h\left(h^{1}\left(S_{A}\right)\right)\\ h^{3}\left(S_{A}\right)=h\left(h^{2}\left(S_{A}\right)\right)\\ \cdots \\ h^{n}\left(S_{A}\right)=h\left(h^{n-1}\left(S_{A}\right)\right) \end{array}\right\}$$ (1)

The resulting sequence of values (i.e., *h*^*1*^*(S*_*A*_*)*, *h*^*2*^*(S*_*A*_*)*, *…*, *h*^*n*^*(S*_*A*_*)*) shown in (1) is the identity hash chain of *A*.

After the hash chain is generated, the authentication entity *A* stores it securely in the form of key-value pairs using a local cryptographic chip, where the number of hash times *i* is the key and the corresponding item *h*^*i*^*(S*_*A*_*)* is the value, and then applies to publish *h*^*n*^*(S*_*A*_*)* on the consortium chain to disclose it to other AEs. Due to the one-way nature of the hash function, *h*^*k-1*^*(S*_*A*_*)* cannot be calculated when only the value of *h*^*k*^*(S*_*A*_*)* is obtained. On the contrary, *h*^*k*^*(S*_*A*_*)* can be easily calculated if the value of *h*^*k-1*^*(S*_*A*_*)* is obtained. Therefore, when *A* receives an authentication request for the first time, it sends *h*^*n-1*^*(S*_*A*_*)* to the requester, and AE that requests *A* for identity authentication can verify the correctness of *h*^*n-1*^*(S*_*A*_*)* by using the value of *h*^*n*^*(S*_*A*_*)* that has been disclosed. After that, *h*^*n-1*^*(S*_*A*_*)* becomes used up and *A* applies to publish *h*^*n-1*^*(S*_*A*_*)* on the consortium chain. In the next identity authentication process of *A*, *h*^*n-1*^*(S*_*A*_*)* will be used to verify *h*^*n-2*^*(S*_*A*_*)* likewise, thus achieving continuous identity authentication based on hash chains.

### 4.2 Selection of parameters related to hash chains

**Fig 5:**
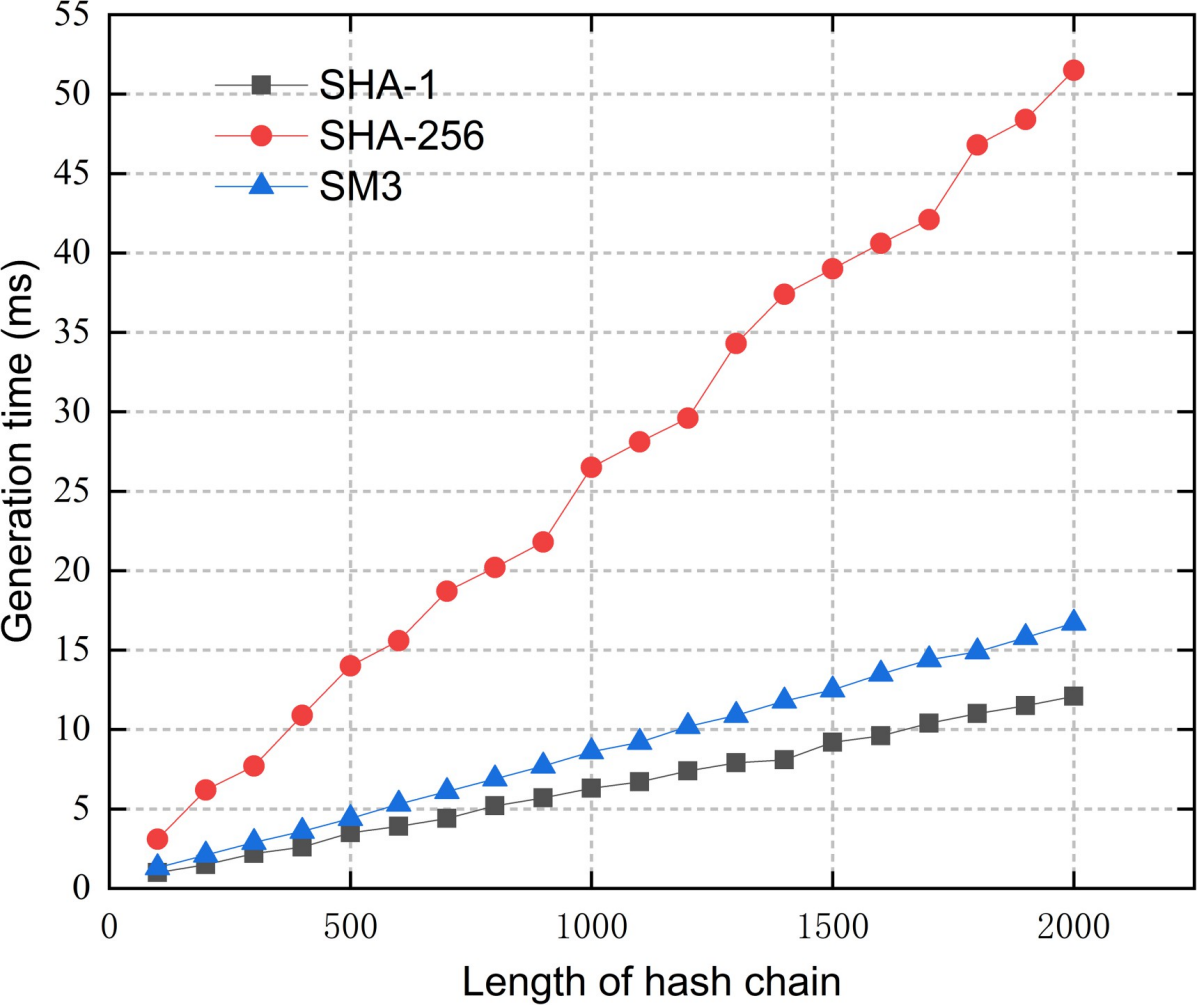
Generation time of hash chains.

**Fig 6:**
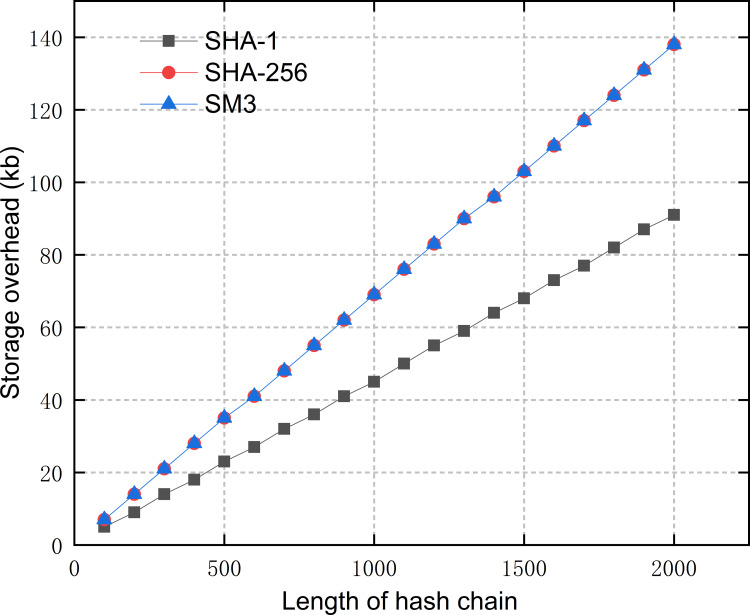
Storage overhead of hash chains.

Since the identity hash chain of an AE is generated at one time and stored locally, both the length of the hash chain and the hash algorithm we use can influence the time and storage overhead of our scheme. Therefore, we compared the time and storage overhead required for generating hash chains of different lengths by three commonly used hash algorithms, SHA-1, SHA-256 and SM3. As it can be seen from Figs 5 and 6, among the three algorithms, SHA-1 has the highest computational efficiency and takes up the least space to store the generated hash chains. The hash chains generated by SHA-256 and SM3 take up the same size of storage space (each item is 256bits), while SM3 does better in terms of efficiency. Therefore, considering both security and resource consumption, we chose SM3 as the generation algorithm of the identity hash chain in the proposed scheme [36]. Then, to limit the hash chain generation time to 10ms, and avoid the renewal process taking too much time to affect the efficiency of authentication, the length of the identity hash chain generated by an AE is chosen to be 1000. Thus, the time overhead required for hash chain generation is averaged to each authentication process, and the additional time consumption for each authentication process is no more than 10ms/1000 = 0.01ms, which is almost negligible. The hash chain regeneration method does not impose a large computational and storage burden on the resource-constrained IEDs of SSCS.

### 4.3 Hash chain renewal mechanism

The identity hash chain of an AE is of finite length and is generated at one-time. When the hash chain is used up, it needs to be regenerated so that the subsequent authentication of the AE can be continued. Considering that third-party key management is not introduced in our scheme, it is not appropriate to use public key cryptographic algorithms, so we introduced a hash chain renewal mechanism based on One-Time Signature (OTS) referring to [37–39].

**Fig 7:**
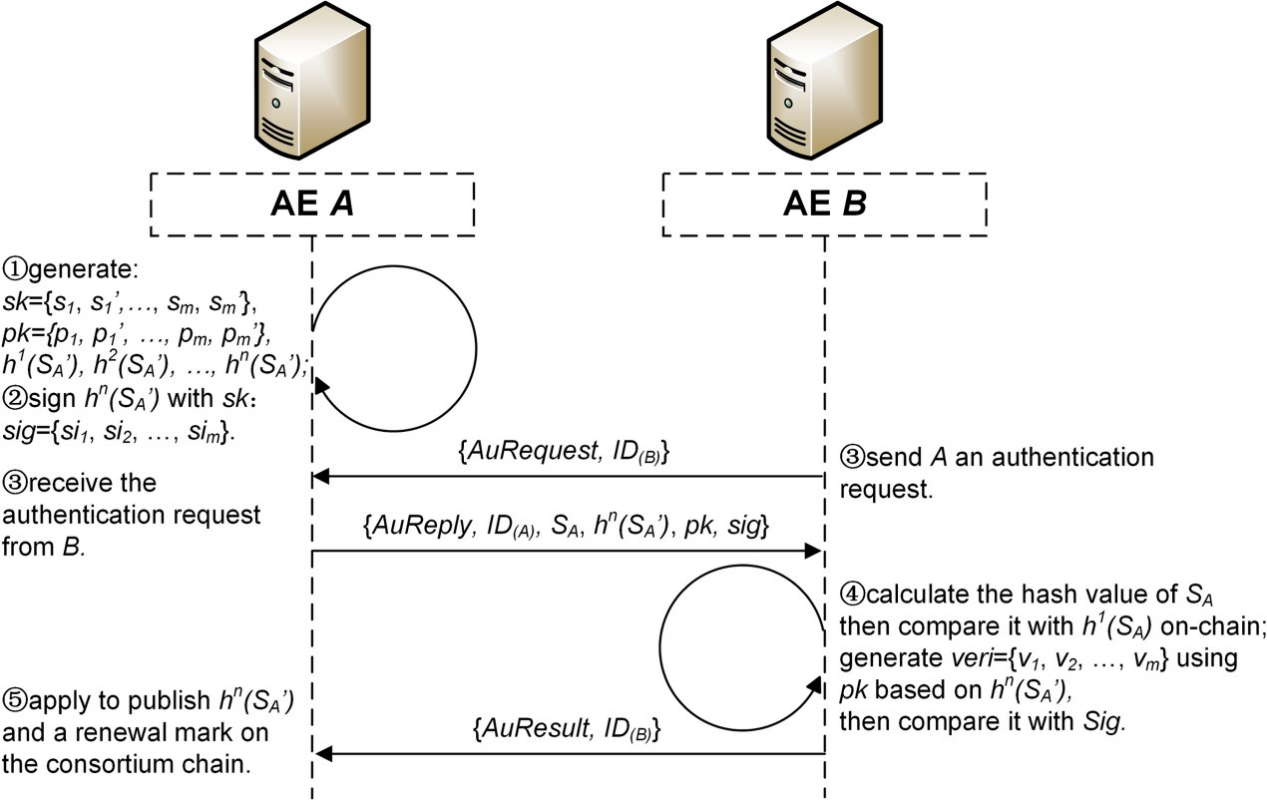
The process of hash chain renewal.

For an AE *A*, when it is repeatedly authenticated until *h*^*1*^*(S*_*A*_*)* is disclosed, the identity hash chain of *A* will be renewed automatically. Steps are detailed as follows (Fig 7).

**Step 1.** *A* first generates a private key set *sk* = {*s*_*1*_, *s*_*1*_*’*, …, *s*_*m*_, *s*_*m*_*’*} and the corresponding public key set *pk* = {*p*_*1*_, *p*_*1*_*’*, …, *p*_*m*_, *p*_*m*_*’*}, where *s*_*i*_ and *s*_*i*_*’* are a pair of random numbers of 128 bits, *p*_*i*_ = *h(s*_*i*_*)*, *p*_*i*_*’* = *h(s*_*i*_*’)*, 1≤*i*≤*m*, *m* = 256, *h()* refers to the hash function selected in Section 4.3.2. Then, *A* chooses a new seed *S*_*A*_*’* and generates a new identity hash chain of length *n*, where *n* = 1000.
**Step 2.** *A* generates an OTS *sig* = {*si*_*1*_, *si*_*2*_, …, *si*_*m*_} using *sk* for *h*^*n*^*(S*_*A*_*’)*, the last item of the new hash chain. The generation rule of OTS is that if the *i*-th bit of *h*^*n*^*(S*_*A*_*’)* is 0, then the value of *si*_*i*_ is set to be equal to the value of *s*_*i*_, otherwise it is equal to the value of *s*_*i*_*’*, where 1≤*i*≤*m* and *m* = 256.
**Step 3.** *A* receives a new authentication request from another authentication entity *B*. Then, *A* sends a reply message *AuReply* = {*S*_*A*_, *h*^*n*^*(S*_*A*_*’)*, *pk*, *sig*} to *B*, where *S*_*A*_ is the seed of the old identity hash chain of *A*.
**Step 4.** After receiving *AuReply*, *B* calculates *h(S*_*A*_*)* and compares the result with *h*^*1*^*(S*_*A*_*)* published on the consortium chain to verify the identity of *A*. Then, according to the same rule as Step 2, *B* generates verification set *veri* = {*v*_*1*_, *v*_*2*_, …, *v*_*m*_} using *pk* based on the value of *h*^*n*^*(S*_*A*_*’)*. *B* calculates *sig’* = {*si*_*1*_*’*, *si*_*2*_*’*, …, *si*_*m*_*’*}, where *si*_*i*_*’* = *h(si*_*i*_*)*, 1≤*i*≤*m*, *m* = 256, and compares *sig’* with *veri*. If *sig’* = *veri*, it can be verified that *h*^*n*^*(S*_*A*_*’)* is an item of the identity hash chain of *A*. If both verifications are passed, *B* sends back *A* a message of authentication success, otherwise *B* sends an alert to MC.
**Step 5.** After receiving the message of authentication success, *A* applies to publish *h*^*n*^*(S*_*A*_*’)* and a renewal mark on the consortium chain.

As it can be seen from Fig 5, the total time for SM3 algorithm to hash 256 numbers is no more than 2.5ms. Therefore, together with the time required for hash chain generation, a hash chain renewal can still be controlled to be completed within 10ms.

## 5. The proposed scheme

### 5.1 Registration phase

**Fig 8:**
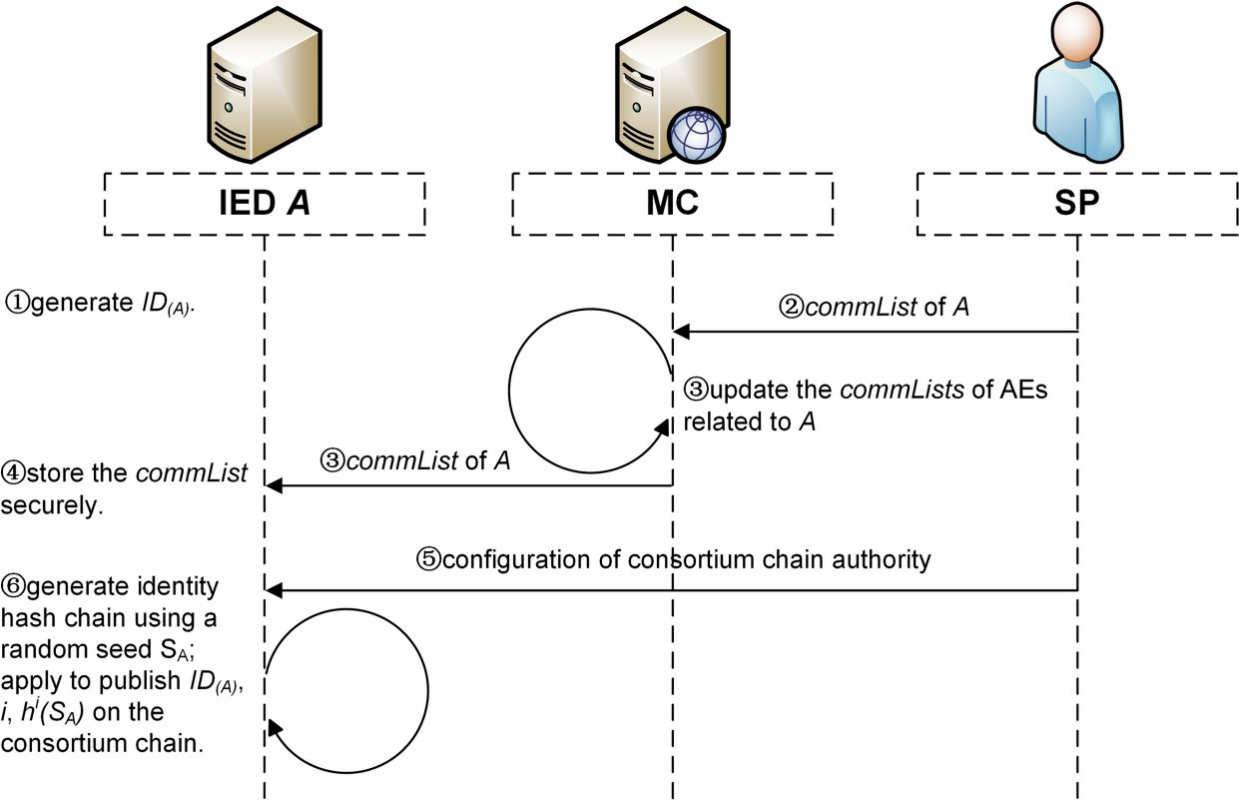
The process of registration.

The registration phase refers to the process of an IED joining the authentication system as an AE. For an IED *A*, the registration process is detailed as follows (Fig 8).

**Step 1.** *A* generates a unique identification, noted as *ID*_*(A)*_.
  Each IED needs a unique identification to conduct operations such as end-to-end communication, marking the published authentication information, querying data on-chain.As shown in Table 2, the unique identification of an AE designed in this scheme consists of two fields, network address and pID. Further, the network address contains IP address and port number, which represents the identity of the AE in the network. The pID represents the physical identity of the AE, which is uniquely assigned, difficult to forge, and traceable throughout the life cycle.

**Table 2:** Fields of the unique identification.

Field		Size
Network address	IP address	4 bytes
	Port number	2 bytes
pID	Company code	1 byte
	Identification code	1 byte
	Serial number	8 bytes
	Check code	1 byte
**Step 2.** SP make a list of information of other AEs that have communication relationship with *A* (hereinafter called the *commList* of *A*), according to the network topology and upload the list to MC.
**Step 3.** MC updates the *commLists* of AEs related to *A* and sends modified parts to them respectively. Then, MC sends the communication list of *A* to *A*.
**Step 4.** *A* receives the communication list and stores securely using a local cryptographic chip.
**Step 5.** *A* joins the consortium chain and obtains the corresponding authority of the chain according to the configuration of SP. If *A* is an IED in a master station, it is configured to become an AuN with the authority to maintain the consortium chain and participate in its consensus process. Otherwise, *A* is configured to be an AcN with only the authority to read data on-chain.
**Step 6.** *A* randomly chooses an integer *S*_*A*_ as the seed and generates the identity hash chain, which is securely stored using a local cryptographic chip. Then, A applies to publish *ID*_*(A)*_, *h*^*i*^*(S*_*A*_*)* and hash chain index *i* on the consortium chain to disclose them to other nodes, where *i* = 1000, and *ID*_*(A)*_, *h*^*i*^*(S*_*A*_*)* and *i* are jointly referred to as an authentication credential.

The registration process performed on an IED is shown in Algorithm 1.

**Algorithm 1** Registration process

**Input:** *netAddr*, *pID*, hashLength *n*

**Output:** *ID*, *commList*, *hashChain*, *isAuthorized*

1: **function Registration (***netAddr*, *pID*, *n***)**

2: *ID* ← **generateID (***netAddr*, *pID***)**

3: *commList* ← **getList ()**

4: **new** List *hashChain*

5: *S* ← **random()**

6: *hashChain* ← **append (***hashChain*, *S***)**

7: **while** *n*>0 **do**

8:  *S* ← **hash(***S***)**

9:  **append (***hashChain*, *S***)**

10:  n ← n-1

11: **end while**

12: **if inMasterStation (***ID***) then**

13:  *isAuthorized* ← **true**

14: **else**

15:  *isAuthorized* ← **false**

16: **end if**

17: **broadcast (***ID*, *S***)**

18: **end function**

### 5.2 Identity authentication phase

#### 5.2.1 The process of identity authentication

After registration and joining the authentication system, identity authentication can be performed between AEs with communication relationship, and the identity authentication process is parallel to their normal services through different communication methods.

**Fig 9:**
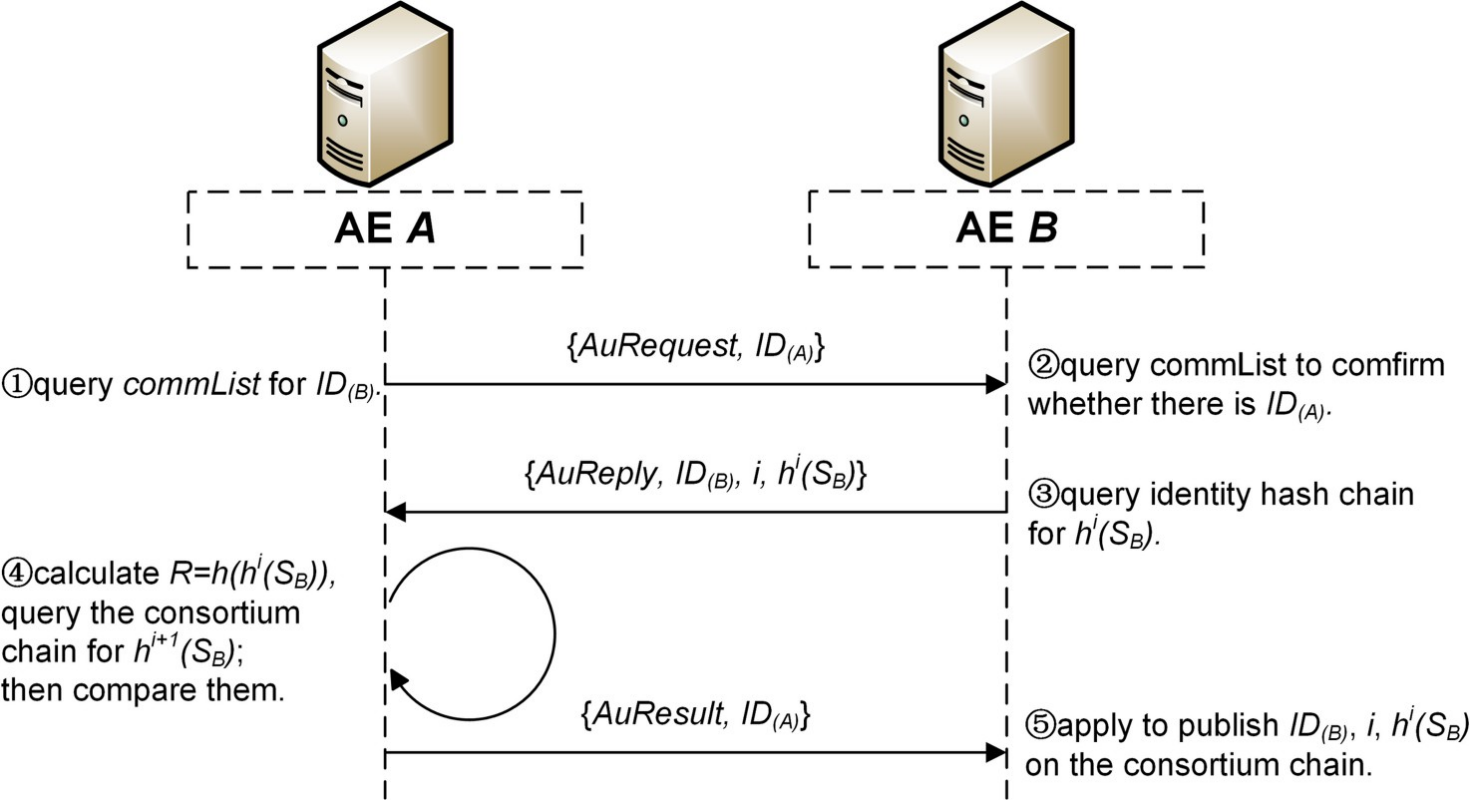
The process of identity authentication.

For two AEs *A* and *B*, when *A* sends an authentication request to *B*, the identity authentication process is detailed as follows (Fig 9).

**Step 1.** *A* obtains the unique identification of *B* by querying the locally stored *commList*, noted as *ID*_*(B)*_, and sends an authentication request to the network address contained in *ID*_*(B)*_, with *ID*_*(A)*_ sent as well.
**Step 2.** After receiving the authentication request, *B* queries its *commList* to confirm whether there is *ID*_*(A)*_. If *ID*_*(A)*_ does exist in the list, the next step is carried out. Otherwise, *B* sends a security alert to MC, which will correct the *commLists* of *A* and *B* according to the actual network topology of SSCS, and the identity authentication process is ended.
**Step 3.** *B* sends the index *i* and *h*^*i*^*(S*_*B*_*)* to *A*, where *i* is the maximum index number of the hash value that *B* has not yet disclosed, the initial value of *i* is 1000 and *h*^*i*^*(S*_*B*_*)* is got by querying the identity hash chain *B* stores locally.
**Step 4.** *A* calculates *R = h(h*^*i*^*(S*_*B*_*))*, and traverses the consortium chain in reverse order to query the value *r* of *h*^*i+1*^*(S*_*B*_*)*. Then, *A* compares the values of *R* and *r*, If *R = r*, A sends back a message of authentication success. Otherwise, *A* sends a security alert to MC and the identity authentication process is ended.
**Step 5.** After receiving the message of authentication success, *B* applies to publish *ID*_*(B)*_, *i* and *h*^*i*^*(S*_*B*_*)* together on the consortium chain.

The identity authentication process performed on the AE that requests for authentication is shown in Algorithm 2.

**Algorithm 2** Identity authentication process

**Input:** *commuList*, *requesterID*

**Output:** *result*

1: **function IdentityAuthentication (***commuList*, *isAuthorized*, *requesterID***)**

2: *receiverID* ← **queryList (***commuList***)**

3: **send (***AuRequest*, *requesterID*, *receiverID***)**

4: *i*, *h*^*i*^**← getReply (***receiverID***)**

5: *r*: **queryChain (***receiverID*, *i+1***)**

6: *R* ← **hash(***h*^*i*^**)**

7: **if** *R*! = *r* ← **then**

8:  **alert ()**

9:  *result* ← **fals**

10: **else**

11:  **send (***AuResult*, *requesterID***)**

12:  *result* ← **true**

13: **end if**

14: **end function**

#### 5.2.2 Timeout mechanism

An AE may send out multiple items of its identity hash chain in succession in response to multiple authentication requests in a short time. For example, when an AE *A* receives authentication requests from three AEs *B*, *C*, and *D* respectively at the same time, let the maximum index number of hash value not yet disclosed by *A* be *k*, and *A* will send authentication information containing *h*^*k*^*(S*_*A*_*)*, *h*^*k-1*^*(S*_*A*_*)* and *h*^*k-2*^*(S*_*A*_*)* to *B*, *C*, and *D* respectively. Before the identity authentication process of *B* is completed, *h*^*k*^*(S*_*A*_*)* will not be disclosed, and *C* cannot get the information needed to continue the authentication (i.e., the value of *h*^*k-1*^*(S*_*A*_*)*) by querying the consortium chain, thus the identity authentication process of C will be blocked. Similarly, the identity authentication process of *D* will also be blocked. Moreover, after the end of identity authentication of *B* and before that of *C* and *D*, the attacker may intercept the values of *h*^*k*^*(S*_*A*_*)* and *h*^*k-1*^*(S*_*A*_*)*, masquerade as *A* and carry out replay attack and masquerade attack to other AEs.

To avoid that, we propose a timeout scheme and apply it to the authentication phase. We make each AE in the proposed scheme maintain an authentication request queue, and all authentication requests are enqueued sequentially in the chronological order they are received. Each AE starts a timer after responding to a request and sending the corresponding authentication information. The preset timeout time is equal to the average time required for an identity authentication process. After the timeout, the AE automatically applies to disclose the hash value used in the current authentication process and starts to respond to the next authentication request in the queue. Due to the relatively fast calculation speed of the hash function, the time required for one authentication is relatively shorter. That is to say, it is difficult for an attacker to finish intercepting the authentication information and forging the identity before the timeout, thus ensuring the continuity of identity authentication and improving its security.

## 6. Security analysis

### Theorem 1

The unique identification can provide traceability of AEs.

*Proof*. The unique identification of an AE consists of its IP address, port number and pID, and is backed up in MC and other related AEs. A legitimate identification uniquely corresponds to a legitimate AE. Additionally, the pID in the identification is associated with the physical location information of the AE. So when an AE misbehaves, MC or other AEs related to it can reveal its identity. SP can also get the location of the suspicious AE through its pID and check whether it is attacked. Hence, the unique identification can provide traceability of AEs.

### Theorem 2

The proposed scheme can resist replay attack.

*Proof*. Let the total time that an attacker A needs to complete the attack be *T*_*A*_. Then *T*_*A*_ = *T*_*1*_ + *T*_*2*_, where *T*_*1*_ is the normal lantency of authentication phase of a legitimate AE *B*, and *T*_*2*_ is the lantency caused by intercepting and resending process of A. However, according to 5.2.2, *B* will disclose *h*^*i*^*(S*_*B*_*)* and make it useless after the average time of the normal latency of authentication phase, which is denoted as *T*_*0*_. Since *T*_*1*_ ≈ *T*_*0*_, it can be inferred that *T*_*A*_ > *T*_*0*_, which means the attack process takes more time than a normal authentication process, and AEs can disclose the current passwords before the replay attack is completed to disable the attack. Hence, the proposed scheme can resist replay attack.

### Theorem 3

The proposed scheme can resist masquerade attack.

*Proof*. Since Theorem 2 is proofed, the attacker A can only conduct masquerade attack by generating the identification and forging a legitimate identity. If A wants to forge the identity of a legitimate AE *B*, A needs to obtain both the identification of *B*, i.e. *ID*_*(B)*_, and the identity hash chain generated by *B*. *ID*_*(B)*_ can be easily stolen or generated. As for the hash chain, however, it is securely stored in a local cryptographic chip on *B*, A cannot easily steal this key information required for authentication by cyber intrusion. Moreover, due to the one-way nature of the hash function, even if A gets the authentication password *h*^*i*^*(S*_*B*_*)* currently used by *B* through eavesdropping or other means, he still cannot calculate *h*^*i-1*^*(S*_*B*_*)* and other undisclosed hash values (namely *h*^*j*^*(S*_*B*_*)*, where 0 < *j* < *i*-1), in which case A cannot masquerade as *B* and pass the subsequent authentication. Hence, the proposed scheme can resist masquerade attack.

### Theorem 4

The proposed scheme can resist DoS attack.

*Proof*. In the proposed scheme, the identity authentication is based on the consortium chain adopting PBFT consensus mechanism. All AuNs participate in the consensus process, and the complete ledger has backups in AuNs. Even if a single node suffers from DoS attack, the distributed authentication system can still keep the normal operation relying on other peer nodes. Additionally, PBFT provides 33% Byzantine fault tolerance, and only when the attacker destroys or hijacks one-third of the nodes in the system, consensus results can be maliciously controlled, which is very hard to achieve in the closed environment of the power system. Consequently, the proposed scheme can resist DoS attack.

## 7. Experiment and analysis

In this section, we designed and implemented multiple sets of experiments, first comparing the execution time of different algorithms and different schemes, then evaluating the performance of the proposed scheme in terms of both time latency and storage, and finally designing two attack scenarios to test the security of the proposed scheme.

We implemented a prototype system in GO language. Compared with mature blockchain platforms such as Hyperledger Fabric, our prototype system is lightweight and functionally customizable and is more suitable for IEDs with limited computing and storage resources. The prototype system is deployed on embedded Linux terminals with Ubuntu 18.04.3 LTS x86 system, Intel(R) Core (TM) i5-6300HQ CPU @ 2.5 GHz processor and 3GB RAM, and its configuration is almost the same as actual IEDs operating in SSCS. Nodes in the prototype system include an MC and several AEs.

### 7.1 Comparison of execution time

The advantage of the proposed scheme is that the computing load on IEDs is low and the computing speed is relatively fast. To further illustrate, we first compared the execution time of RSA, ECDSA and SM3 algorithms for 128-byte data, with execution time of RSA and ECDSA referring to time for a complete process of encryption and decryption. As shown in Table 3, it can be seen that the execution time of RSA and ECDSA operations is far more than that of SM3. That is to say, compared with the identity authentication methods based on mainstream public-key cryptographic algorithms, hash chain-based authentication is more efficient and more suitable for IEDs with limited computing resources.

**Table 3:** Execution time of different algorithms at different operation times.

Operation Times	RSA-2048	ECDSA	SM3
**400**	695 ms	870 ms	2 ms
**800**	1398 ms	1750 ms	4 ms
**2000**	3443 ms	4301 ms	10 ms

Since there is no known scheme that applies blockchain to the authentication of IEDs in SSCS, schemes proposed by the related works also have different process with ours and our proposed scheme lacks a scheme that can be directly and clearly compared. Then, we conducted a simple evaluation of the efficient of our proposed scheme and those of [13–18] from a theoretical perspective. [13] is a message authentication method protocol that improved traditonal PKI-based scheme, and [14–18] are similar authentication protocols based on ECC algorithm which is more efficient than other public key algorithms. Omitting operations that have little influnce on the system performance, our scheme takes 1000*T*_*hash*_ in registrtion phase and 1*T*_*hash*_ in authentication phase, where *T*_*hash*_ represents the time latency of hash operations. The total time of the authentication process of [13] is more than 5*T*_*hash*_+14*T*_*HMAC*_, where *T*_*HMAC*_ represents the execution time of a HMAC function and *T*_*HMAC*_≈300*T*_*hash*_ according to the simulation result in [13]. So the execution time of [13] is more than 4205*T*_*hash*_, which is much more than that in our scheme. Similarly, after comparison, the total time of the authentication process of protocols in [14–18] is more than 12*T*_*hash*_+4*T*_*SM*_, where *T*_*SM*_ represents the execution time of ECC Scalar multiplication and *T*_*SM*_≈218*T*_*hash*_ according to [15]. Thus the authentication time of [14–18] is more than 884*T*_*hash*_, which is similar to the total execution time in our scheme, but is much more than our authentication time. As we can see from the analysis, our proposed scheme is very simple and the computation cost is also relatively low.

### 7.2 Time latency analysis of the authentication process

As can be seen from Section 2.5, the total time required for the identity authentication phase can be detailed as follows:

$$T=T_{comm}+T_{q}+T_{hash}$$ (2)

*T* represents the time latency of the entire identity authentication phase. *T*_*comm*_ represents the time latency of communication, which is caused by the transmission of authentication requests and responses in the network and is related to network conditions. *T*_*q*_ represents the time latency of on-chain data query, which is related to the node scale. *T*_*hash*_ represents the time latency of hash operations, which is the time overhead of hashing the authentication password and comparing it with the disclosed hash value. As shown in Table 3, the process of hash operations takes little time.

**Fig 10:**
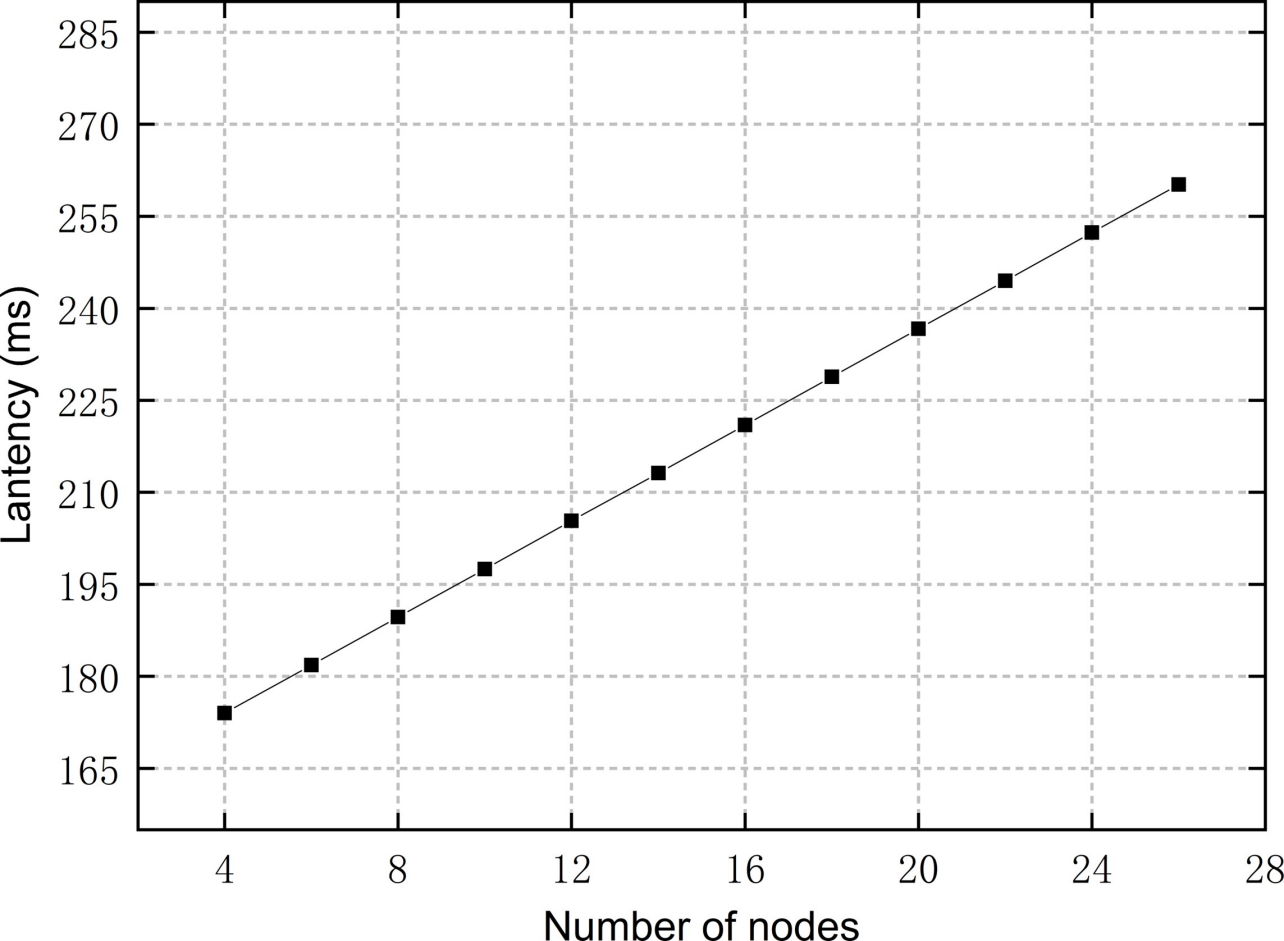
Latency of authentication at different numbers of nodes.

We tested the time latency of the identity authentication phase, and the results are shown in Fig 10. It can be seen that as the number of nodes increases, *T*_*q*_ increases with a slow growth rate, thus also causing *T* to increase slowly. Before the node scale gets large enough, *T*_*comm*_ accounts for the majority of *T*, while *T*_*comm*_ is just over 165 ms. The number of IEDs with authentication requirements in SSCS is no more than 100. According to Fig 10, then, we can infer that the increase in *T*_*q*_ will not exceed 350 ms in practical application, and *T* will not exceed 550 ms, which can meet the authentication requirements of IEDs without bringing much additional time latency.

### 7.3 Storage overhead analysis

**Fig 11:**
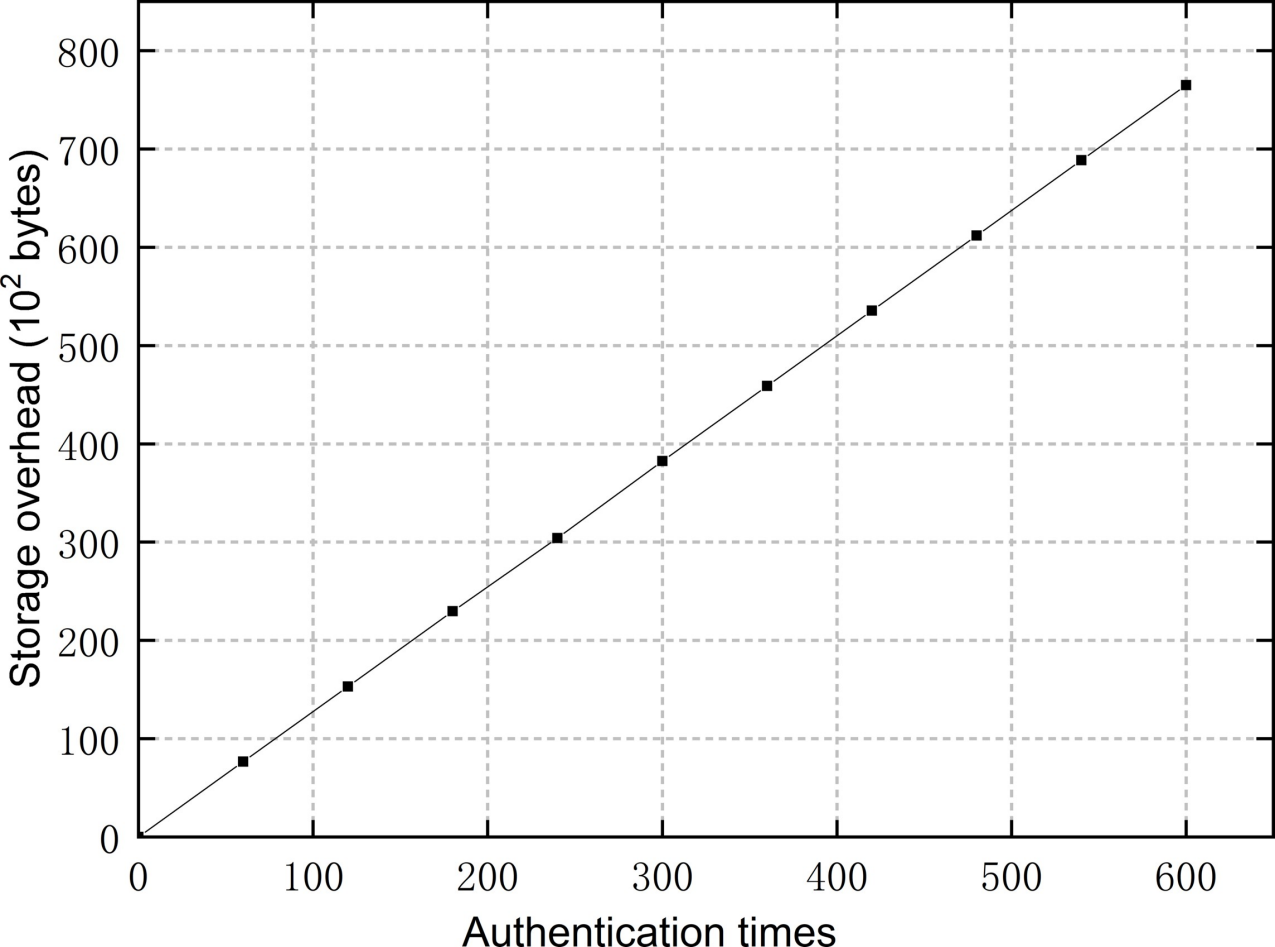
Storage overhead at different authentication times.

The consortium chain in our scheme is applied for storing disclosed authentication credentials that have been previously used in the identity authentication phase. Obviously, at a certain number of nodes, the size of the consortium chain ledger is related to the times of identity authentication. We tested the storage overhead of the consortium chain, where the total number of nodes is set to ten and each node is set to continuously send authentication requests at an interval of 20 seconds to related nodes in the order of its *commList*. As shown in Fig 11, the size of the ledger is proportional to the times of authentication. In the proposed scheme, the size of the block header is 80 bytes according to Table 1, and the size of an authentication credential is 51 bytes, where the unique identification takes 17 bytes according to Table 2, the hash index takes 2 bytes and the hash value corresponding to the index takes 32 bytes. When each block stores only one credential, 1M space can hold more than 7500 blocks. Additionally, the authentication among IEDs is conducted at an interval of more than 20 seconds in practice, and the capacity of an IED is no less than 8G. Consequently, the authentication scheme is able to continuously operate for a long time while bringing affordable storage overhead to IEDs.

### 7.4 Security validation

We tested the effects of the timeout threshold and the number of malicious nodes on the proposed scheme by setting two attack scenarios respectively and validated the security of the proposed scheme.

#### Scenario 1

**Fig 12:**
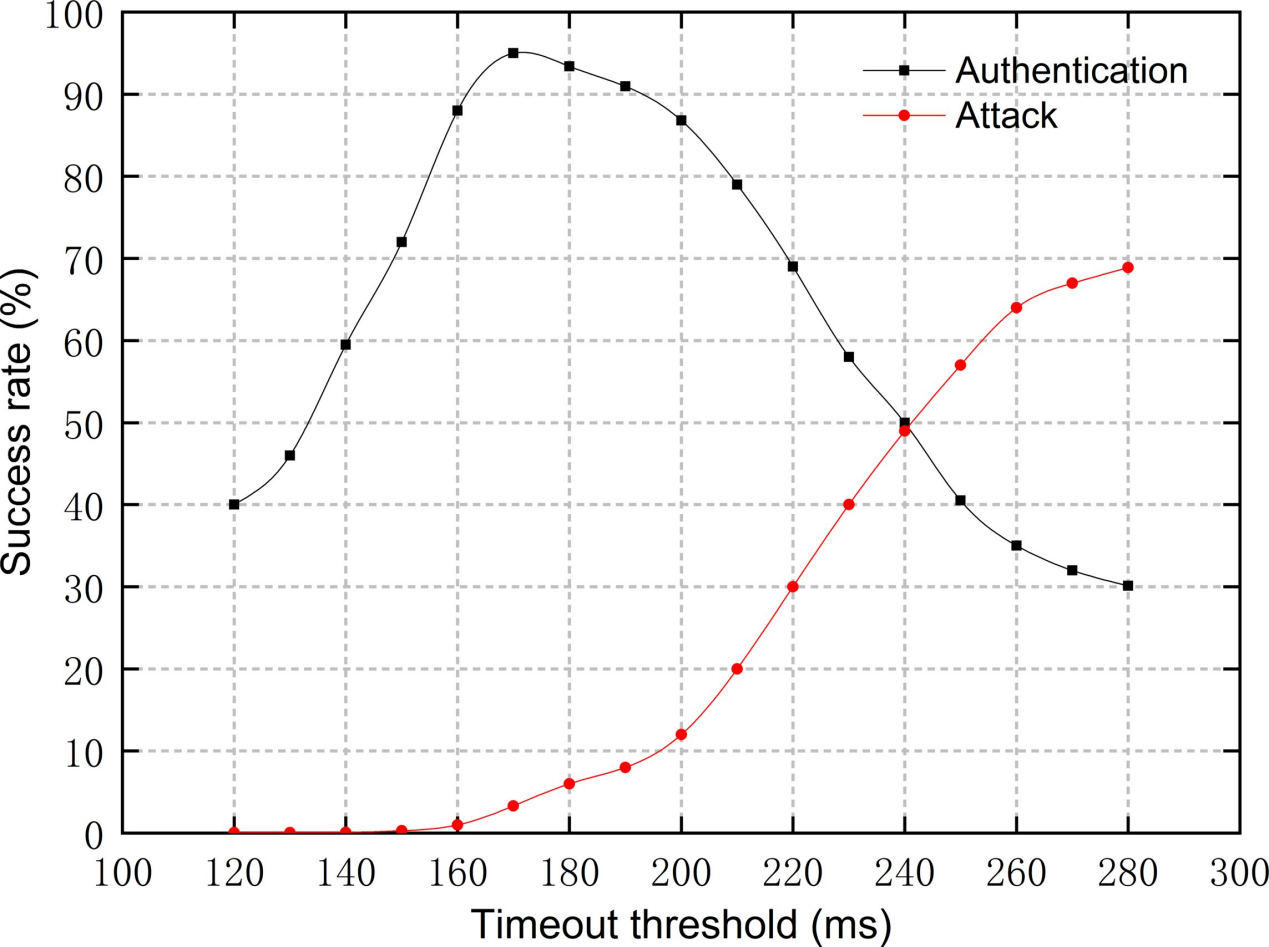
Authentication success rate and attack success rate at different timeout thresholds under scenario 1.

We set the number of AuNs to four and let two AEs *A* and *B* send authentication requests to each other at an interval of 20 seconds. A malicious node *C* established a connection with *A* and *B*, respectively, and tried to carry out a replay attack to deceive *A* or *B*. We tested the average time required for the identity authentication phase in this scenario, which was 160 ms. Then, we set up a timeout mechanism as described in Section 2.5.2, and specified that the next authentication process was blocked until the current one was completed. We tested the effects of timeout threshold on authentication success rate and attack success rate, the results of which are shown in Fig 12. It can be seen that a low timeout threshold will limit the success rate of both authentication and attack, causing both to increase as the timeout threshold increases. However, when the timeout threshold exceeds a certain value, the authentication success rate will start to be greatly affected by the attack success rate, and will gradually drop to below 40% as the timeout threshold increases. In other words, if no timeout mechanism is set, the system will be extremely vulnerable to attacks. When the timeout mechanism is set up and the threshold is set to the average time required for the identity authentication phase, the success rate of replay attack is extremely low and the authentication success rate exceeds 90%, and the impact is within the acceptable range. The results prove that the timeout mechanism proposed in this paper can effectively resist replay attacks, thus improving the security of SSCS.

#### Scenario 2

**Fig 13:**
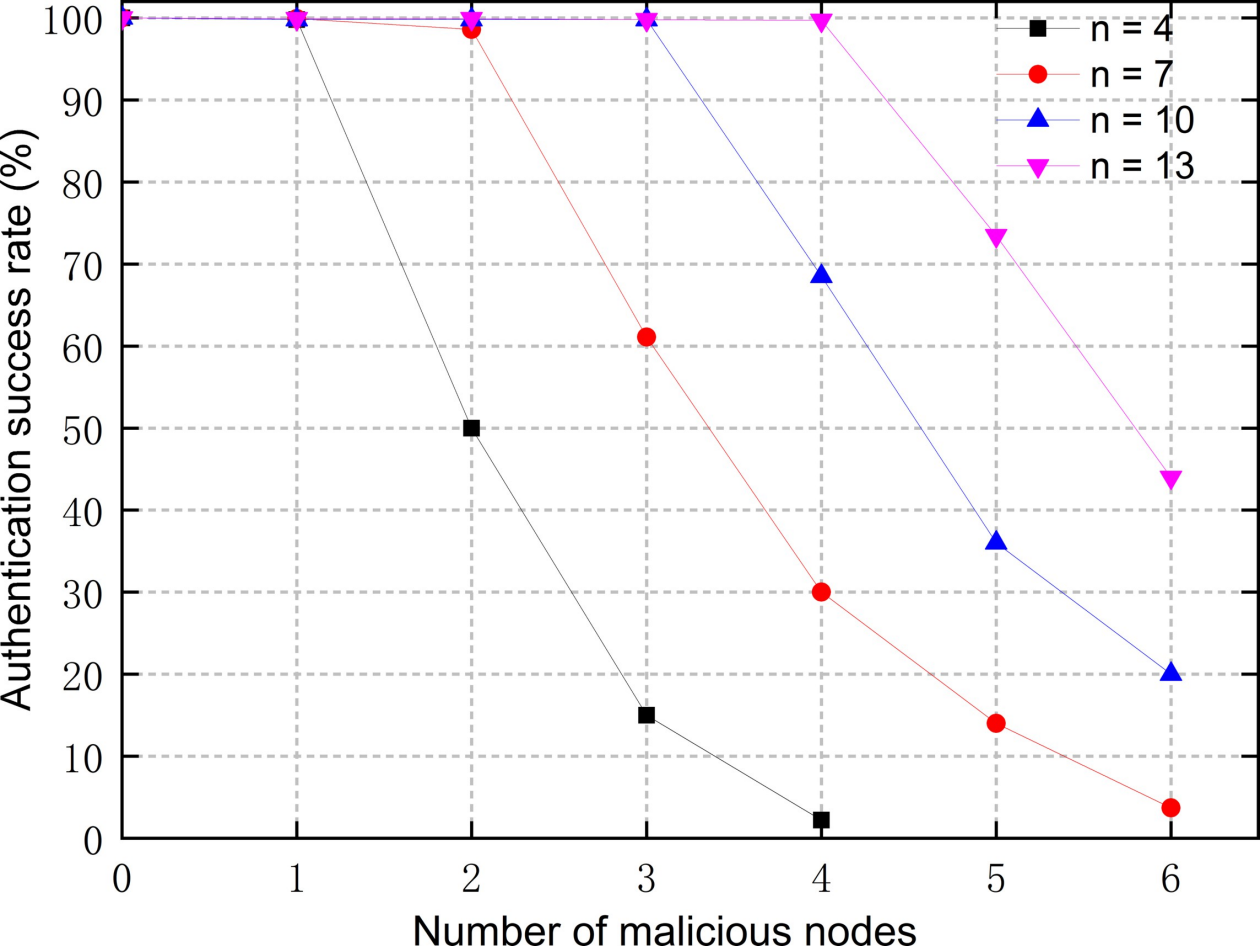
Authentication success rate at different numbers of malicious nodes under scenario 2.

The number of AuNs was set to be *n*, while among them the number of malicious nodes was set to be *f*. The malicious nodes were set to randomly send either normal messages, error messages, or no messages during the process of consensus. Fig 13 shows the effect of the number of malicious nodes on authentication success rate. As can be seen, when the number of malicious nodes is less than 1/3 of the number of AuNs, i.e., *n* > 3*f*, identity authentication processes can be completed normally and are almost unaffected. But when *f* exceeds 1/3 of *n*, malicious nodes become able to significantly interfere with the process of consensus, thus making it difficult for blocks to be generated and for authentication credentials to be published on the consortium chain. That results in a rapid decrease in the authentication success rate. However, in the actual working scenario of IEDs, the closed environment of the power system makes it very hard for attackers to control more than 1/3 of at the same time. Attackers are more likely to carry out DoS attacks to disable several AuNs, which can be tolerated according to test results in Fig 13. Consequently, the proposed scheme can resist DoS attacks to some extent.

## 8. Conclusion

At present, the lack of security protection mechanism among IEDs of SSCS exposes the power system to lots of cyber security risks. In this paper, we propose a lightweight identity authentication scheme for IEDs of SSCS based on blockchain technology. We combine the IP address and port number with pID as the unique identification of IEDs, which can represent both network identity and physical identity with full life-cycle traceability of an IED. By applying blockchain to ensure the tamper-proof of authentication credentials and to realize decentralized identity authentication to replace the centralized PKI-based authentication mechanism commonly used in the power system and replace the use of public and private keys with hash values as one-time authentication passwords, thus eliminating the overhead of deploying trusted third parties for key management and the time overhead of key generation. Security analysis shows that the proposed scheme can resist replay attack, masquerade attack and DoS attack to a certain extent, and it also enables the traceability of malicious behaviors of IEDs. Experiments show that in the proposed blockchain-based authentication scheme for IEDs of SSCS, the time latency of the identity authentication phase can be limited within 550ms, and the storage overhead is also little. That is to say, the computing and storage resources of IEDs will not be excessively occupied and their normal communication of services will not be affected.

The proposed scheme is mainly designed for the working scenario of IEDs of SSCS in China, but it can be seen as a reference for authentication schemes for other resource-constrained systems as well. As for future work, we plan to extend the application scenario to make it more general for other resource-constrained systems. Additionally, since our scheme has not yet solved the storage expansion problem brought by the growing blockchain, we will further study and then introduce the deletion mechanism of blockchain to our scheme. Moreover, there are still points for improvement in the security of the proposed scheme. We need to make it more rigorous.

## Supporting information

S1 File (ZIP)
